# SPR-DETR: DETR with Self-Supervised Learning and Position Relation Modeling for UAV-Based Catenary Support Component Detection in Electrified Railways

**DOI:** 10.3390/s26103077

**Published:** 2026-05-13

**Authors:** Tao Liang, Zhigang Liu, Linjun Shi, Haonan Yang, Ning Ma, Hui Wang

**Affiliations:** School of Electrical Engineering, Southwest Jiaotong University, Chengdu 610097, Chinahut19960710@163.com (H.Y.);

**Keywords:** deep learning, object detection, catenary support components (CSCs), self-supervised learning

## Abstract

Catenary support components (CSCs) are essential for the safe and efficient operation of electrified railway systems. However, detecting CSCs in images presents significant challenges due to the scarcity of labeled data, the presence of complex and diverse backgrounds, and the difficulties associated with multi-scale variations. To tackle these issues, this paper introduces a novel detection framework designed explicitly for CSCs. First, a Siamese-based self-supervised learning framework is designed as a pre-training strategy to reduce the reliance on labeled data, effectively leveraging unlabeled images and significantly lowering annotation costs. This pre-training approach enables the model to focus on identifying and extracting relevant features from prior knowledge, honing its ability to discern key patterns and structures within the data. Second, the Vision Attention-based Intrascale Feature Interaction (Vision-AIFI) and Relation Vision Module (RVM) are proposed to enhance the model, which can strengthen its ability to extract multi-scale features and effectively address challenges posed by complex backgrounds and scale variations. Third, a Dempster–Shafer (DS) evidence theory-based detection head is inserted to improve classification confidence and localization precision, ensuring accurate detection results in complex inspection scenarios. Finally, a UAV-based dataset for CSCs is constructed and validation experiments are performed. To evaluate the model, we used several standard COCO metrics, including mAP (77.84), APs (67.84), APm (70.31), and APl (90.04). In addition, the framework is further evaluated for Domain Generalization, which can demonstrate its strong adaptability and high detection accuracy for real-world CSC detection tasks.

## 1. Introduction

During train operation, the mechanical contact between the pantograph and the catenary system frequently induces severe vibrations of the catenary support and suspension devices, resulting in failures such as loosening, damage, or breakage of the catenary support components (CSCs) [1,2,3,4]. If these issues are not addressed promptly, they may threaten the safe operation of the train and even result in serious rail accidents [5]. Ensuring the structural integrity of these systems is also vital for the broader resilience and security recovery of complex traffic networks [6]. Historically, catenary inspections were predominantly executed through manual methodologies, which are labor-intensive, time-consuming, and susceptible to safety hazards [7,8,9]. The subjective nature of human judgment frequently results in inconsistent and inaccurate inspection outcomes [10,11]. Automated inspection technologies such as 4C inspection vehicles have been widely adopted to address these limitations, offering significant improvements in detection efficiency and precision [12]. Nevertheless, these systems’ substantial expense and constrained adaptability impede their extensive implementation [13]. Consequently, a recent focus has been unmanned aerial vehicle (UAV)-based inspection methods as a cost-effective and adaptable alternative [14]. By equipping UAVs with industrial-grade cameras, which feature high resolution, high dynamic range, durability, high frame rates, and low latency, they can effectively perform large-scale area monitoring and frequent, rapid detection tasks, playing a crucial role in CSC detection [15]. Furthermore, advanced multi-view UAV technologies are increasingly driving diverse applications, ranging from complex action recognition to industrial inspections [16]. Despite these advancements, ensuring accurate and reliable detection remains challenging due to complex backgrounds and multi-scale variations captured in UAV images, highlighting the importance of robust detection algorithms.

To address these challenges, catenary support component detection has evolved into two main categories: traditional methods and deep learning-based methods. In traditional approaches, Yang et al. proposed a method for classifying shafts and insulators using the Hough transform and affine invariant moments (AIMs) to identify insulator porcelain bottles [17]. Han et al. used Scale Invariant Feature Transform (SIFT) for detecting fractures in rotary double ears through feature point matching and boundary curve analysis [18]. Xu et al. combined LBPHOG features with a Support Vector Machine (SVM) to detect rotation binaural components, outperforming LBP or HOG features alone [19]. Zhong et al. combined SIFT, improved Random Sample Consensus (RANSAC), and Hough transform to detect defective swivel clevis pins, using grayscale analysis of length ratios for accurate detection [20]. Zhang et al. used DHOG features and discrete cosine transform (DCT) for auxiliary catenary wire loss fault detection, improving accuracy through feature enhancement and arc detection [21]. Traditional detection methods typically follow a two-step process: feature extraction using algorithms like Hough transform [22], SIFT [23], or HOG [24], followed by classifiers like SVM [25] for defect detection. These methods, however, struggle with complex scenarios, such as diverse backgrounds and multi-scale targets, and are limited by reliance on handcrafted features, making generalization across different datasets difficult [26,27]. With the rise of deep learning, these methods have gained prominence, learning features directly from data, thus eliminating the need for handcrafted feature design [28,29]. Deep learning methods are generally divided into two-step detection and one-step detection. Guo et al. proposed an improved Faster R-CNN for detecting droppers in overhead contact systems (OCSs), using a balanced attention feature pyramid network (BAFPN) for multi-scale feature fusion and center-point rectangle loss (CR Loss) for improved bounding box regression [30]. Liu et al. introduced a fault detection system for loose strands in isoelectric lines using Faster R-CNN and Markov random field models for pixel segmentation and defect identification [31]. Liu et al. used an improved Faster R-CNN for detecting brace sleeve screws in catenary systems, addressing challenges in high-resolution and low-resolution proposal maps [32]. Tan et al. proposed a Mask RCNN-based method for detecting insulator defects using vertical projection and K-means clustering for precise segmentation [33].

Compared to two-stage detectors that rely on proposal-based frameworks, single-stage detectors simplify object detection by directly predicting bounding boxes and class probabilities from feature maps, offering faster inference speeds [34]. For example, Zhang et al. employed YOLOv3 combined with an Out-of-Distribution Detector for Neural Networks (ODIN) to detect catenary dropper states, effectively mitigating localization errors [35]. Guo et al. proposed an unsupervised learning method using TPH-YOLOv5 to enhance low-light catenary images, achieving improved localization accuracy [36]. Similarly, Chen et al. developed a fault diagnosis framework for current-carrying rings, utilizing spatial attention and channel weight maps in an improved RetinaNet model to enhance defect detection performance [37]. Recently, DEtection TRansformer (DETR) and its variants have emerged as powerful alternatives, utilizing bipartite matching and global self-attention mechanisms to eliminate handcrafted components. Alongside DETR, recent cutting-edge paradigms, such as Mamba-based architectures featuring multi-receptive fields or graph-guided mechanisms [38,39], as well as spike-based brain-inspired neural networks [40], have also been explored to push the boundaries of anchor-free CSC detection on UAVs. Despite their success, generic DETR models fundamentally lack specific inductive biases for dealing with the extreme scale variations and highly cluttered backgrounds typical of UAV top-down perspectives.

Despite these advancements, existing methods primarily focus on isolated tasks, such as object localization or defect detection, and lack comprehensive designs to handle the challenges posed by UAV-based catenary inspections. UAVs capture images from a top-down perspective, which inevitably include complex backgrounds, such as bridges, tracks, and surrounding vegetation. These intricate environments complicate object detection and make distinguishing relevant components from irrelevant background features difficult [41]. Additionally, catenary support components (CSCs) present significant multi-scale variations, with some being very small, further exacerbating detection challenges [42]. While standard self-attention mechanisms facilitate interaction between features, they primarily rely on absolute 2D position encoding and do not explicitly model the relative spatial configurations and geometric relationships between target boxes. These issues often lead to misalignment between localization and classification, reducing the effectiveness of general detection algorithms.

Moreover, the reliance on manually annotated data in deep learning-based methods presents another critical limitation. Annotating large-scale datasets for UAV-captured images is labor-intensive, time-consuming, and prone to inconsistencies. As a result, the scarcity of labeled data hinders the development of more robust and adaptable detection frameworks [43]. To overcome this, self-supervised learning (SSL) frameworks have gained traction by leveraging unlabeled data for pre-training. For instance, masked image modeling (MIM) has recently been adapted for self-supervised pre-training on catenary support components to effectively mitigate data scarcity [44]. However, existing generic SSL methods are not tailored for the specialized structural characteristics of CSCs. Addressing the challenges of UAV-based catenary inspections requires innovative approaches that enhance feature representation for complex backgrounds and multi-scale objects while reducing reliance on extensive labeled datasets. This paper proposes a comprehensive detection framework incorporating advanced methods to tackle these issues to ensure accurate and reliable catenary support component (CSC) detection under diverse and complex conditions. Specifically, the contributions of this paper are listed as follows.

(1) A self-supervised learning framework with prior-enhanced pre-training is proposed to alleviate the reliance on labeled data and enhance the feature extraction.
(2) The Relation Vision Module (RVM) and AIFI module with position relation embedding are combined to strengthen the spatial relationship modeling and optimize multi-scale feature fusion for improved detection performance.
(3) A Dempster–Shafer (DS) evidence head is developed to enhance classification confidence and localization precision, which can ensure robust performance under diverse conditions.
(4) A comprehensive UAV-based dataset for CSC detection is constructed to support the training and evaluation in real-world scenarios.

The following content is structured as follows: Section 2 introduces the proposed method. Section 3 mainly introduces the experimental environment and settings, datasets, experimental results, and analysis. Section 4 presents the conclusions and further prospects.

## 2. The Proposed Method

**Figure 1:**
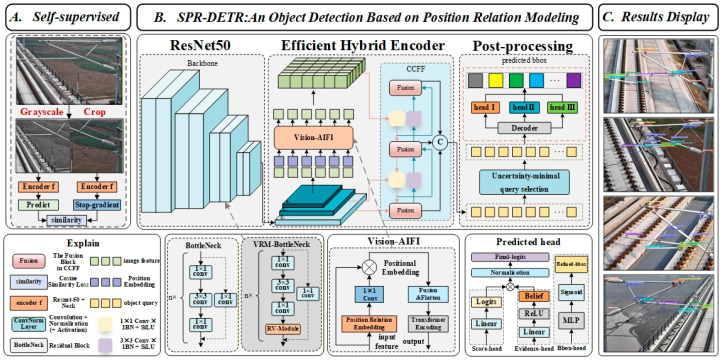
The structural diagram of SPR-DETR. SPR-DETR mainly consists of VRM Bottleneck, Vision-AIFI and DS.

This paper proposes an end-to-end detector based on positional relationships for efficient detection of all catenary support components. This section presents the architectural diagram of the proposed method, as illustrated in Figure 1. The framework comprises three principal components: data processing, the SPR-DETR main structure, and the result display. Among the components, the VRM Bottleneck plays a pivotal role in the ResNet-50 network, extracting and optimizing multi-scale image features. Relation-AIFI within the encoder section embeds positional relationships and achieves feature fusion. On the other hand, the DS evidence head constitutes a component of the detection header, integrating evidence information to generate accurate object detection results.

### 2.1. Self-Supervised Learning with Multi-View Feature Alignment (MVSSL)

A self-supervised learning framework is designed to address the reliance on extensive labeled datasets for CSC detection, drawing inspiration from recent advancements in frameworks such as SimSiam [45] and SimMIM [46]. These methods have demonstrated the ability to leverage large volumes of unlabeled data to pre-train models, significantly reducing the need for manual annotations [47]. The framework of our method generates multiple variations of the same input image by employing diverse data augmentation techniques such as flipping, rotation, grayscale conversion, and scaling [48]. These augmented views are treated as alternative representations, enabling the model to learn robust and invariant features through feature alignment [49]. The backbone of the framework employs a Siamese-based network, where the encoder extracts high-level features from augmented views. A feature alignment mechanism ensures consistency across these augmented views, effectively simulating the diversity of real-world scenarios. This process significantly enhances the model’s ability to generalize under complex and variable conditions while reducing reliance on labeled data during training.

**Figure 2:**
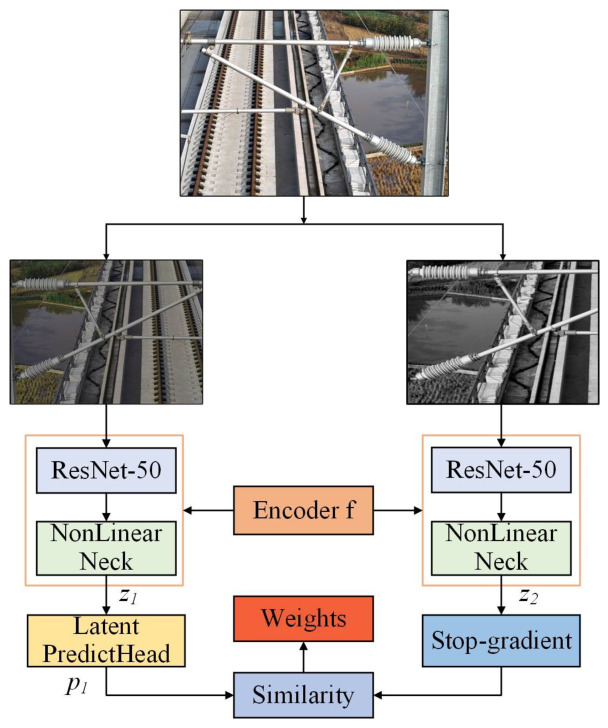
The structural diagram of self-supervised learning.

As illustrated in Figure 2, the generation of two distinct views, designated *x*_1_ and *x*_2_, of the input image is initiated by random data augmentation. An encoder network with shared weights subsequently processes these views. The encoder comprises a backbone network and a projection layer, which extract high-level features from the views to obtain feature maps *z*_1_ and *z*_2_, respectively.

(1) $$\begin{array}{c} z_{1}=Encoder\left(x_{1}\right)\\ z_{2}=Encoder\left(x_{2}\right) \end{array}$$

To improve model robustness, Gaussian noise is added after data augmentation to simulate real-world uncertainty and reduce sensitivity to input disturbances. This helps the model learn more noise-tolerant features. The Gaussian noise is formulated as follows:

(2) $$\begin{array}{c} {x_{1}}^{\prime }=x_{1}+N\left(0,\sigma^{2}\right)\\ {x_{2}}^{\prime }=x_{2}+N\left(0,\sigma^{2}\right) \end{array}$$

where N(0, *σ*^2^) represents Gaussian noise with mean 0 and variance *σ*^2^, applied to the input images *x*_1_ and *x*_2_. We chose a noise standard deviation (*σ* = 0.01). The goal is to introduce a moderate amount of noise to enhance the model’s generalization ability while avoiding disruption of the key features in the input data. A smaller noise standard deviation helps prevent overfitting and preserves the essential information in the data, ensuring that the model learns robust feature representations.

To extract global features at varying scales in an efficient manner, an adaptive average pooling operation is incorporated into the encoder network. This process transforms the feature map into a fixed-size global representation, enabling the model to retain more helpful information across various resolutions. The pooled features are listed as follows:

(3) $$\begin{array}{c} z_{1}^{\prime }=AdaptiveAvgPool2d(z_{1})\\ z_{2}^{\prime }=AdaptiveAvgPool2d(z_{2}) \end{array}$$

Subsequently, through a nonlinear prediction head, the model maps the features of one view to the feature space of the other. The predictor is structured as a multi-layer perception (MLP), and the predicted results *p*_1_ and *p*_2_ correspond to the feature predictions of *z*_1_′ and *z*_2_′, respectively. These predicted features are employed to calculate the feature similarity and alignment loss function. In the design of the loss function, a cosine similarity-based metric is initially employed to maximize the feature similarity of analogous views, as it is scale-invariant, is computationally efficient, and helps avoid gradient vanishing, ensuring stability during training [50]. This is achieved through the application of the following formula:

(4) $$Loss_{\cos}=1-\frac{z_{1}^{\prime }\cdot z_{2}^{\prime }}{{\left\|z_{1}^{\prime }\right\|}_{2}{\left\|z_{2}^{\prime }\right\|}_{2}}$$

where *z*_1_′ and *z*_2_′ are the feature vectors after pooling; **·** represents the dot product; and ||·|| represents the L2 norm. This loss function aims to maximize the feature similarity between similar views. To further improve the effect of feature matching, this paper also introduces the Wasserstein distance into the loss function. The Wasserstein distance [51] measures the difference in distribution between the predicted features and the target features by calculating the Manhattan distance (L1 distance) between the features, as follows:

(5) $$\begin{array}{l} D_{\mathrm{wasserstein}\;}\left(z_{1}^{\prime },p_{1}\right)=\frac{1}{N}\sum_{i=1}^{N}\left|z_{1}^{\prime (i)}-p_{1}^{\left(i\right)}\right|\\ D_{\mathrm{wasserstein}\;}\left(z_{2}^{\prime },p_{2}\right)=\frac{1}{N}\sum_{i=1}^{N}\left|z_{2}^{\prime (i)}-p_{2}^{\left(i\right)}\right| \end{array}$$

Through this self-supervised learning framework, the model is trained on a drone catenary dataset and pre-trained weights are obtained. These pre-trained weights provide a strong foundation for the object detection task, significantly improving detection performance. The effectiveness of this method not only is reflected in feature learning but also demonstrates good generalization ability in downstream tasks.

In order to simultaneously achieve feature alignment during the self-supervised pre-training stage and effectively address the distribution uncertainty in UAV-captured scenarios, we combine the mutual information maximization criterion with probabilistic distribution distance metrics. The total loss function (*L_total_*) for self-supervised pre-training is defined as follows:

(6) $$L_{total}=L_{inv}(C)+\lambda \cdot L_{red}(C)$$

Among them, *L_inv_* denotes the Invariance Loss, which aims to enhance the representation consistency of the model across different augmented views; (*L_red_*) represents the Redundancy Reduction Loss, designed to prevent feature degradation; and (λ) is a hyperparameter that balances the two loss terms, which is set to 0.0051 in the experiments of this paper.

The core of this loss function lies in the construction of the Adjusted Cross-correlation Matrix (***C***). Different from conventional similarity metrics, we dynamically couple cosine similarity with the Wasserstein distance:

(7) $$C=\frac{1}{1+\gamma \cdot D_{W}(\hat{z},z)}\cdot \left(\frac{{\hat{z}}^{⊤}z}{N}\right)$$

In this formula, $\left(\frac{{\hat{z}}^{⊤}z}{N}\right)$ characterizes the sample-level alignment strength between the prediction feature *z* and the target feature z. We introduce an adaptive scaling factor $\omega =1/(1+\gamma \cdot D_{W})$ based on the Wasserstein distance *D_W_*, where γ denotes the distance weight coefficient.

The physical implication of this design is as follows: when the model perceives a significant deviation between the predicted distribution and the target distribution (i.e., high uncertainty), the value of *D_W_* increases and the scaling factor *_W_* decreases, thereby automatically reducing the weight of the corresponding sample in the total loss. With this mechanism, SPR-DETR can suppress noise interference caused by UAV motion blur and occlusion, and learn more robust feature representations. The specific expressions of the invariance loss *L_inv_* and redundancy reduction loss *L_red_* are defined as follows:

(8) $$\begin{array}{l} L_{inv}=\sum_{i}{\left(1-C_{ii}\right)}^{2},\;\;\\ L_{red}=\sum_{i}\sum_{j\neq i}C_{ij}^{2} \end{array}$$

### 2.2. Relation Vision Module Based on Grouped Residual Self-Attention

The Relation Vision Module (RVM) enhances the model’s spatial perception ability, leveraging advancements in self-attention mechanisms and geometric feature modeling. Inspired by the effectiveness of self-attention in capturing dependencies across regions, the RVM combines self-attention with geometric information fusion to better model the spatial relationships inherent in image features [52,53]. This design approach aligns with recent research emphasizing the integration of geometric priors in enhancing object detection performance.

**Figure 3:**
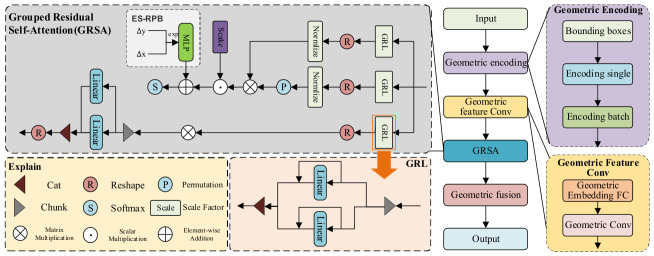
The structural diagram of the Relation Vision Module based on Grouped Residual Self-Attention.

The module introduces a Grouped Residual Self-Attention (GRSA) mechanism, which effectively captures the spatial relationships between regions by processing grouped features [54]. This mechanism optimizes computational efficiency while retaining the ability to process complex spatial interactions. Figure 3 illustrates the structure of the RVM, highlighting its modular design and the integration of GRSA. This architecture focuses on refining feature representations for subsequent processing stages, contributing to robust object detection in varied environments. The input to this module is a feature matrix of the form X ∈ ℝ*^B×N×C^*, where B is the batch size, N is the number of regions, and C is the feature dimension of each region. Initially, the input features are transformed linearly to generate a query (Q), a key (K), and a value (V), respectively. These three entities then calculate their relationships through a self-attention mechanism, thereby adjusting the weight values of the input features. In the implementation, the query, key, and value are calculated using the following linear transformation: the understanding of objects’ spatial structure through the weighted fusion of geometric features.

(9) $$Q=W_{Q}X,\;K=W_{K}X,\;V=W_{V}X$$

where *W*_Q_, *W*_K_, and *W*_V_ represent the learned weight matrices, whereas Q, K, and V denote the results obtained by performing a linear transformation on the input features. The attention matrix, Attn (Q, K), is then calculated by computing the dot product between Q and K.

(10) $$Attn(Q,K)=\frac{QK^{T}}{\left\|Q\right\|\left\|K\right\|}$$

The attention matrix illustrates the interrelationship between the characteristics of a given region and those of other regions. By assigning a value of V to the attention matrix, a self-attention weighted feature representation is ultimately derived. Traditional self-attention mechanisms suffer from high computational complexity. The GRSA module addresses this issue by introducing the Grouped Residual Layer (GRL), which processes Q, K, and V through a grouping mechanism. Specifically, the input features are split along the channel dimension, and two independent linear layers are used to generate Q, K, and V. This grouping reduces computational overhead while preserving the self-attention mechanism’s ability to capture inter-region relationships. As a result, the GRSA module significantly improves computational efficiency. In practical implementation, this process is expressed by the following equation:

(11) $$Q=L_{GRL_{Q}}(X),K=L_{GRL_{K}}(X),V=L_{GRL_{V}}(X)$$

Subsequently, GRSA integrates the calculated attention matrix with the relative position encoding (Exponential-Space Relative Position Bias, ES-RPB) to further refine the modeling of spatial relationships. The relative position bias is processed by a small multi-layer perceptron (MLP), which ensures that the model focuses more closely on the relationship between adjacent pixels while simultaneously reducing the impact of noise. In particular, the position bias is determined through the application of the following formula:

(12) $$\begin{array}{l} \Delta X=\mathrm{sign}(\Delta X)\cdot (1-\exp(-|\alpha \cdot \Delta X|)),\;\\ \Delta Y=\mathrm{sign}(\Delta Y)\cdot (1-\exp(-|\beta \cdot \Delta Y|)) \end{array}$$

(13) $$B_{ES-RPB}=MLP(\Delta X,\Delta Y)$$

where α and β are trained distance sensitivity factors that control the sensitivity of relative positions; the position deviation information is incorporated into the attention matrix, which is calculated using self-attention to ultimately derive the weighted output. In the Relation Vision Module, the GRSA module captures the spatial relationships between regions through this optimized self-attention mechanism and incorporates the geometric information of the target box. By calculating the geometric characteristics of the target box, including its center coordinates, width, and height, and encoding them into a geometric feature vector, these features are then weighted and integrated with the output features of the GRSA module.

(14) $$\begin{array}{c} \delta_{x}=log\left(\left|\frac{{\;center\;}_{x}-{\;center\;}_{x}^{\prime }}{w}\right|\right)\\ \delta_{y}=log\left(\left|\frac{{\;center\;}_{y}-{\;center\;}_{y}^{\prime }}{w}\right|\right)\\ \delta_{w}=log\left(\frac{w}{w^{\prime }}\right),\delta_{h}=\log\left(\frac{h}{h^{\prime }}\right) \end{array}$$

The bounding box’s four coordinates (*x*_1_, *x*_2_, *y*_1_, and *y*_2_) are input into the geometric coding module to generate spatial perception features. The center coordinates of the bounding box (centerx, centery) are calculated and compared with the center coordinates of other boxes to ascertain the spatial difference between the boxes. Subsequently, the difference values are scaled and logarithmically transformed, thereby yielding four geometric coding features (*δ*_x_, *δ*_y_, *δ*_w_, and *δ*_h_).

Once the geometric feature encoding has been obtained, the features undergo further processing via convolution to extract more profound spatial information. The objective of the convolution is to extract local spatial relationships from the geometric features, thereby enhancing the model’s comprehension of the spatial relationships between objects. The convolution layer employs a sliding window to locally process features, thereby enabling the capture of local spatial relationships between object boxes. The resulting geometric features after convolution processing are represented by C, as illustrated below:

(15) $$C=Conv\left(\tanh\left(Linear\left(\delta_{x},\delta_{y},\delta_{w},\delta_{h}\right)\right)\right)$$

where C represents the geometric features, is generated and then integrated with the output of the GRSA module through a weighted summation. The GRSA module employs a self-attention mechanism to capture the spatial relationships between regions, while the geometric features offer more explicit spatial positioning information. The following formula can express the weighted fusion process:

(16)  O =GRSA_output⊙(C⊕1)

where the GRSA_output is a feature calculated by the GRSA module that represents the relationship between regions and spatial information, and ⨀ indicates an element-wise multiplication operation, while ⨁ indicates an extended dimension. The two sources of information are combined through weighted fusion to form a comprehensive feature representation that incorporates both spatial perception and geometric structure. The fused feature, designated as O, represents a feature map that integrates the spatial location of the target box with the object relationship.

The feature map offers more precise spatial comprehension, facilitating more accurate object recognition and reconstruction by the subsequent image reconstruction module. In conclusion, the features will be conveyed as input to subsequent modules, enabling the model to achieve more precise object detection and image reconstruction in complex spatial environments.

### 2.3. Improving Intra-Scale Feature Interaction with Position Relation Embedding

The AIFI module [55] primarily addresses the interaction between co-scale features, yet it does not explicitly model the relative spatial configuration between target boxes. While the interaction between co-scale features through the self-attention mechanism facilitates capturing the relationship between regions, the AIFI module does not sufficiently model the relative positional relationship between target boxes.

**Figure 4:**
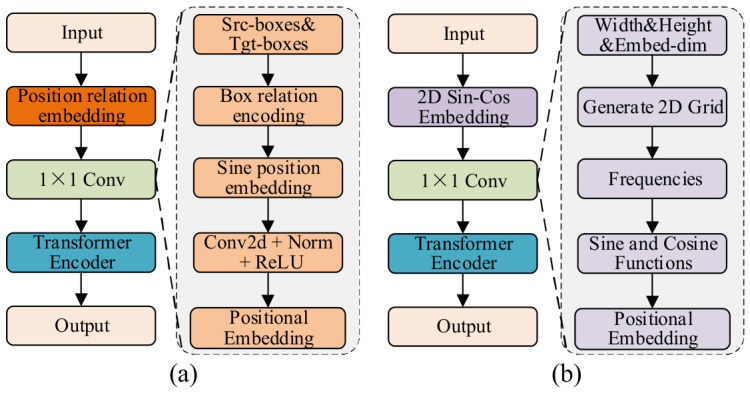
(**a**) The structure diagram of Relation-AIFI; (**b**) the structure diagram of AIFI.

The AIFI module furnishes absolute positional data for the feature map using 2D cosine position encoding, as illustrated in Figure 4b. However, it does not explicitly model the relative spatial configuration between target boxes. The formula is as follows:

(17) $$Pos(x,k)=\sin\left(\frac{x}{T^{2k/d}}\right),Pos(x,k+1)=\cos\left(\frac{x}{T^{2k/d}}\right)$$

where x denotes the coordinate of the feature map, and T represents the temperature factor, which controls the change in frequency due to the scale of the embedding. d refers to the encoding dimension, and k is the frequency index used to control the frequency change in the cosine function. This formula generates a cosine embedding for the horizontal and vertical coordinates of the feature map, respectively, which are then spliced together for feature encoding. This method captures the absolute position information of the feature map grid, as opposed to the relative spatial relationship between the target boxes.

Figure 4a illustrates the introduction of a position relation embedding [56] designed to more accurately model the relative positional relationships between target boxes. The positional relationship coding enhances the model’s spatial perception ability by explicitly computing the spatial relationship between each pair of target boxes. Specifically, the spatial relationship between each pair of boxes is constructed by calculating the relative coordinate difference and the width and height ratio. The relative positional relationship between each pair of target boxes, represented as a four-dimensional vector [57], is as follows:

(18) $$e\left(b_{i},b_{j}\right)=\left[\begin{array}{c} \log\left(\frac{\left|x_{i}-x_{j}\right|}{w_{i}}+1\right),\log\left(\frac{\left|y_{i}-y_{j}\right|}{h_{i}}+1\right),\\ \log\left(\frac{w_{i}}{w_{j}}\right),\log\left(\frac{h_{i}}{h_{j}}\right) \end{array}\right]$$

where *x* and *y* represent the central coordinates of the target box, while *w* and *h* indicate the dimensions of the box in terms of width and height; by calculating the relative position information, the relative geometric differences between each pair of target boxes can be obtained.

For each pair of boxes (*b*_i_, *b*_j_), the results of the calculation of e(*b*_i_, *b*_j_) are organized into a relationship matrix E ∈ ℛN × N × 4, where N represents the number of boxes, and 4 represents the four relative position features of each pair of boxes. The matrix can store the spatial relationship between each pair of boxes and subsequently generate a high-dimensional embedding representation.

(19) $$E(i,j)=e\left(b_{i},b_{j}\right)$$

To augment the expressiveness of spatial relationships, we transform the relationship matrix *E* into a high-dimensional embedding. This metamorphosis employs a sinusoidal cosine encoding method, as elucidated by the following equation:

(20) $$\begin{array}{c} Embed(E,2k)=\sin\left(\frac{sE}{T^{2k/d_{re}}}\right),\;\\ Embed(E,2k+1)=\cos\left(\frac{sE}{T^{2k/d_{re}}}\right) \end{array}$$

where the dimension of the position relationship embedding, denoted as *d*_re_, serves to determine the dimension of the embedding space. The scaling factor, denoted as *s*, is employed to adjust the scale of the position relationship encoding, thus affecting the distribution within the high-dimensional space. Using these formulas, the relative position relationships of each pair of boxes are mapped to a high-dimensional space, thus enabling effective modeling of these position relationships within the self-attention mechanism.

Following these transformations, the final embedding shape is *N* × *N* × 4*d*_re_, whereby the relationship between each pair of frames is mapped to a high-dimensional vector representation. Subsequently, the encoded relationship embedding is subjected to a linear transformation to obtain the final relationship weights. The relationship weight, denoted as Rel(bi, bj), is calculated using the following formula:

(21) $$Rel\left(b_{i},b_{j}\right)=\max\left(\varepsilon ,W\cdot Embed\left(b_{i},b_{j}\right)+B\right)$$

where W and B represent the learned weights and biases, respectively. The term ε denotes a small constant that serves to guarantee that the relationship weights are consistently positive. This is done to prevent gradient vanishing when they are integrated into the self-attention mechanism.

The generated relationship weight matrix, denoted Rel (bi, bj), has a shape of *N* × *N* × *M,* where M denotes the number of attention heads. These weights are employed in the self-attention mechanism to modulate the interaction between disparate target boxes, thereby facilitating the model’s comprehension of the spatial relationships between target boxes in the target detection task.

A high-dimensional embedding representation is explicitly assigned by introducing positional relationship encoding to capture the relative positional relationships between each pair of target boxes. This approach improves the expressiveness of spatial relationships, enhancing the model’s spatial awareness in complex scenes. It effectively overcomes the limitations of traditional positional encoding techniques, enabling more nuanced modeling of spatial relationships and providing richer prior information for subsequent self-attention calculations, thereby boosting object detection performance.

### 2.4. DS Evidence Head (DS-Head)

**Figure 5:**
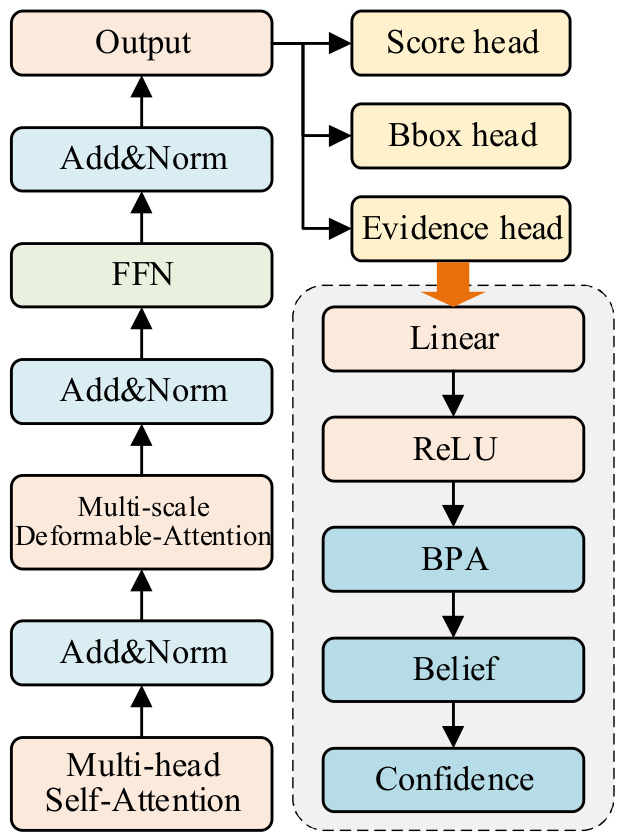
The structure diagram of the DS evidence head.

In our object detection framework, we employ DS evidence theory to enhance the network’s ability to distinguish between target categories [58], especially when dealing with targets that have overlapping or unclear boundaries, as shown in Figure 5. Specifically, an evidence head is designed alongside the classification head of the decoder, working in parallel to generate evidence information for each object [59]. The role of the evidence head is to compute an evidence value for each object category based on the decoder’s output feature map. This evidence value reflects the confidence level associated with the category and captures any uncertainty during object detection. In our investigation, the set of all possible hypotheses, denoted by *X*, comprises all categories. The probability distribution for hypothesis A, denoted by *m*(A), satisfies the following:

(22) $$m(\emptyset )=0,\;\sum_{A\subseteq X}m(A)=1$$

where *m*(A) denotes the level of evidence supporting hypothesis A. Belief represents a significant indicator based on BPA, indicating the credibility of the evidence supporting a hypothesis. The BPAs of all subsets contained within A must be summed to obtain the belief associated with hypothesis set A. The calculation formula is as follows:

(23) $$Bel(A)=\sum_{B\subseteq A}m(B)$$

For each class within the network, a belief value can be calculated based on the output evidence, representing the degree of confidence associated with that class. By calculating the belief of each class, it is possible to more effectively address instances of uncertainty regarding the target, particularly in cases where there are duplicates within the class or where the target information is ambiguous. The belief is calculated by the following formula:

(24) $$Belief(A)=\frac{m(A)}{\sum_{B\in X}m(B)}$$

The confidence level associated with hypothesis set A is calculated by aggregating the BPAs of all its constituent subsets. The evidence head generates an evidence value for each target category by processing the output features of each decoder layer. The evidence value is ensured to be non-negative through the ReLU activation function, which is then used as input for the BPA calculation. This evidence values the network’s confidence in each target category, effectively capturing the uncertainty in target discrimination, mainly when boundaries are unclear or categories overlap. Specifically, the evidence value is computed using the following formula:

(25) $$evidence_{c}=ReLU\left(W_{e}\cdot X+b_{e}\right)$$

where the evidence value of the category, represented by the variable evidence, is multiplied by the weight and bias values of the linear layer, represented by We and be, respectively. This result represents the features of the decoder output.

The principal function of the evidence head is to generate an evidence value for each category that reflects the credibility of the category in question. Subsequently, the evidence values are normalized and converted into a basic probability assignment (BPA). The BPA indicates the degree of support for a given category and reflects the extent to which the model is confident. The normalized BPA formula is given as follows:

(26) $$BPA(c)=\frac{{\;evidence\;}_{c}}{\sum_{c^{\prime }\in C}{\;evidence\;}_{c^{\prime }}}$$

where BPA(c) represents the basic probability distribution of category c, and C denotes the set of all categories.

The evidence values generated by the evidence head provide a corresponding trust metric for each target category, assisting the model in assessing the reliability of different categories. These values serve as a basis for discrimination during the target detection process and significantly improve the robustness of detection, especially in situations involving uncertainties such as complex backgrounds. By incorporating the DS evidence head, the model can dynamically assign trust values to each object category, enabling a more precise evaluation of category reliability. This approach enhances the model’s discriminative capability and improves its ability to handle complex background scenarios, leading to more accurate detection in uncertain environments.

## 3. Experiments and Result Analysis

### 3.1. Experimental Settings

The operating environment of the experimental is Pytorch (version 2.3.1), and the operating environment configuration is as follows: (1) processor: Intel(R) Xeon(R) CPU E5-2697A v4 @ 2.60 GHz; (2) operating memory: 16 G RAM; (3) GPU: NVIDIA RTX 2080TI GPU; (4) code operating environment: Torch = 2.3.1, Python = 3.10, and Ultralytics8.2; (5) CUDA version: CUDA 11.6, cuDNN 8.9.7. The corresponding threshold of non-maximum suppression is set to 0.7 in the following experiments. Next, we use Adaptive Moment Estimation (Adam) as the optimizer to train the network, and the specific parameters of Adam are shown in Table 1. Additionally, the optimizer uses a momentum value of 0.9 and a weight decay of 1 × 10^−4^ to prevent overfitting. In addition, to train the model more efficiently, we perform the following operation: (1) the learning rate is linearly increased from 1 × 10^−4^ to 1 × 10^−3^ during the first 2000 iterations, allowing the model to stabilize in the early stages of training; (2) momentum is gradually increased from an initial value of 0.8 to 0.9 during the first phase of training. In MVSSL, geometric augmentation is applied using RandomResizedCrop with a scale range of [0.2, 1.0] [41] and RandomHorizontalFlip. Color augmentation is implemented via ColorJitter with strengths of {brightness: 0.4; contrast: 0.4; saturation: 0.4; hue: 0.1}, applied with a probability of 0.8, as well as RandomGrayscale with a probability of 0.2. Blurring augmentation [9] is performed using a Gaussian kernel with a standard deviation in the range of [0.1, 2.0].

### 3.2. Introduction of Dataset

**Figure 6:**
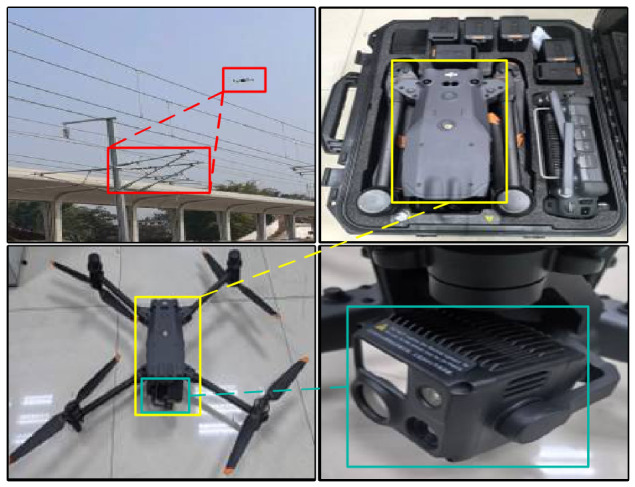
The visualization of the inspection UAV.

A CSC dataset taken by a UAV was constructed to verify the effectiveness of the proposed method. The dataset was collected using a DJI Matrice M30T UAV (DJI, Shenzhen, China) on a railway section. The UAV has a 480,000-pixel zoom camera, a 120,000-pixel wide-angle camera, a laser-ranging sensor, and an RTK navigation and obstacle avoidance system. When capturing images on the line using drones, tests showed that the 20× zoom lens, when used from approximately 50 m away from the contact network, can fully capture all components of the contact network. The detailed hardware configuration is summarized in Table 2, and all other acquisition parameters were kept at default settings. The image of the UAV is shown in Figure 6.

The dataset includes a total of 5832 UAV cruise images, and this experiment studied parts of important electrified railway catenary support components. The dataset constructed in this paper covers a variety of typical road and scene conditions in high-speed railway operating environments. It not only includes conventional open plain areas but also specially integrates complex geographical scenarios such as tunnel entrances and exits, bridge crossing sections, and deep cutting sections. By collecting inspection images under diverse backgrounds, the dataset ensures the model’s detection capability when facing varied ground object features (e.g., vegetation, ballast, bridge structures, etc.), thereby improving the object discrimination ability of SPR-DETR in complex environments along railway lines.

**Figure 7:**
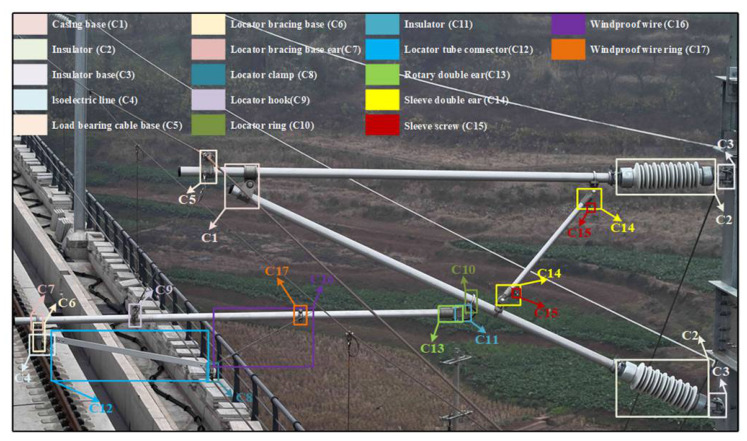
Key label display of catenary support components.

**Figure 8:**
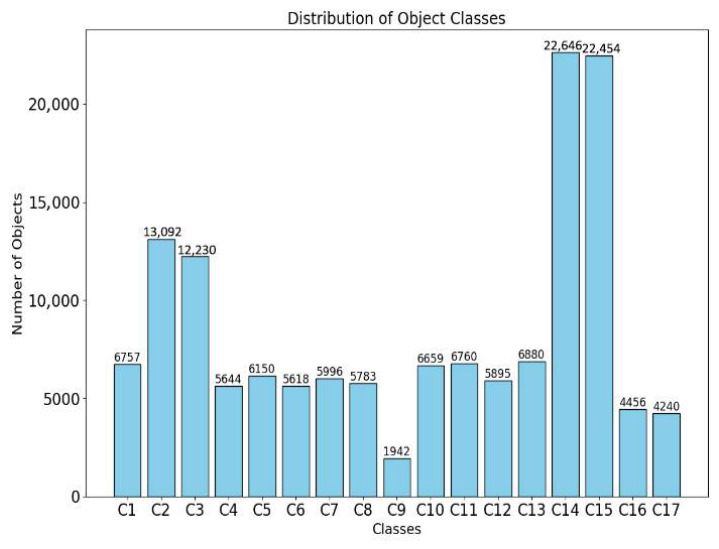
The distribution statistics of UAV dataset.

Specifically, it contains a total of 17 catenary components as shown below: casing base (C1), insulator (C2), insulator base (C3), isoelectric line (C4), load bearing cable base (C5), locator bracing base (C6), locator bracing base ear (C7), locator clamp (C8), locator hook (C9), locator ring (C10), locator tube connector (C11), rectangular locator (C12), rotary double ear (C13), sleeve double ear (C14), sleeve screw (C15), windproof wire (C16), and windproof wire ring (C17). Among them, a total of 143,202 component instances are included, and the distribution of each component is shown in Figure 7 and Figure 8. We divided the training and test sets in a 4:1 ratio.

### 3.3. Procedure of Model Training

The algorithmic steps of the proposed model are listed in Algorithm 1.
**Algorithm 1:** The proposed SPR-DETR Detector***Input:*** The training and testing catenary component images.***Step-1:*** The input RGB image is normalized with the following parameters: (1) the mean value (mean) is [138.96, 135.67, 133.72]; (2) the standard deviation (std) is [60.76, 60.17, 60.65].***Step-2:*** When image size is set to 640, the shorter side is adjusted to 640, and the longer side is scaled proportionally, maintaining the original aspect ratio without exceeding 640.***Step-3:*** The processed image is passed through the backbone network, including convolutional layers and RVMBottleneck modules, to extract multi-scale features. This produces {C3, C4, C5, C6} feature maps, which are fused into {P3, P4, P5, P6} for detection heads.***Step-4:*** The encoder processes features derived from the backbone, applies position relation embedding through Relation-AIFI, and transmits the encoded features to the decoder.***Step-5:*** The encoder outputs are processed by the classification head, regression head, and DS evidence head to predict bounding boxes, classification scores, and evidence parameters, which are then returned.***Step-6:*** During training, the model is optimized by minimizing the combined loss of classification and regression.***Output:*** After training, the optimized model weights are saved for inference, with checkpoints regularly saved to allow for model restoration at any point.

### 3.4. Evaluation Indicator

To reasonably evaluate the detection performance of the model, the following indexes are used in this paper: average precision (AP), mean average precision (mAP), frames per second (FPS), floating-point operations (FLOPs), and parameters (Pa).

(27) $$\begin{array}{c} P=\frac{TP}{TP+FP},R=\frac{TP}{TP+FN}\\ AP=\int \begin{array}{c} 1\\ 0 \end{array}P(R)dR,mAP=\frac{\sum_{q=1}^{Q}AP(q)}{Q} \end{array}$$

where TP, FP, and FN represent the number of the true positive samples, false positive samples, and false negative samples, respectively. P and R represent precision and recall. AP is average precision, which is the area of the PR curve. mAP represents mean average precision, which is the mean value for all catenary components.

### 3.5. Experimental Analysis

***(1) Ablation study analysis:*** In addition, ablation experiments were conducted using the proposed method, including ablation experiments on the Relation Vision Module, Positional Relation Embedding, DS evidence head, and self-supervised learning with multi-view feature alignment. The experimental results are shown in Table 3. FPS was tested using images with a size of 640 × 640. While all other modules were evaluated using the full labeled dataset, MVSSL was tested using only one-third of the labeled data (1555 training images and 389 test images).

The analysis of Table 3 leads to the following conclusions: (1) Through the *T*-test analysis between SPR-DETR with all methods incorporated and the different added methods (mainly analyzing the mAP metric), the T-statistic values are calculated to be in the range [5.70, 30.69] and the *p*-values are in the range [0.0004, 0.018]. Since the *p*-value is less than 0.05, it indicates that SRP-DETR has a statistically significant advantage. (2) The Relation Vision Module (RVM), position relation embedding (PRE), and DS evidence head (DS-head) are demonstrated to markedly enhance the efficacy of the original RT-DETR model. (3) Regarding accuracy, each module is shown to result in the following improvements: the RVM enhances mAP50-95 percentiles to 0.699, the PRE further augments this to 0.727, and the DS-head improves it to 0.735. Upon loading all modules, the maximum mAP50-95 reached is 0.778. (4) Regarding detection speed, the overall inference speed is 23.2 FPS, and the proposed module introduces a negligible additional computational cost, thereby maintaining high efficiency. (5) By employing MVSSL, we achieved effective object detection performance using only one-third of the labeled data, reducing the annotation workload by approximately 66% and significantly lowering data preparation costs.

In conclusion, the proposed module and method achieve an appropriate and effective balance between accuracy and inference speed.

***(2) The analysis of comparative experiments:*** To effectively validate the effectiveness of the proposed method, we conducted comparative experiments with some self-supervised learning methods and classical detection methods, as shown in Table 4 and Table 5. In addition, some experimental conditions were as follows: (1) the prediction results of all experiments were averaged over five random training and test runs; (2) images of size 640 × 640 were used for training and testing. In order to compare the various methods, we performed the following experiments: (1) for the single-step detection methods, we used YOLOv10, YOLOv8 and ATSS as benchmarks; (2) for the two-step detection methods, we used cascaded R-CNN and dynamic R-CNN as benchmarks; (3) for the family of DETR methods (SOTA), we used Align-DETR, DDQ (DETR), DINO, and HDINO methods for comparison experiments.

From the analysis of Table 4, the following conclusions can be drawn: (1) through the T-test analysis between SPR-DETR and the comparison methods (mainly analyzing the mAP metric), the T-statistic values were calculated to be in the range [6.44, 23.14] and the *p*-values were in the range [0.0001, 0.002]. Since the *p*-value is less than 0.05, it indicates that SRP-DETR has a statistically significant advantage. (2) Compared with CNN-based detectors such as SSD and Faster R-CNN, SRR-DETR not only offers stronger global perception and a more unified architecture but also achieves a better balance between speed and accuracy through its end-to-end design and efficient inference pipeline, demonstrating superior performance especially in complex environments and small-object detection tasks. (3) Our method achieves optimal performance in mAP, AP50, AP75, APs, and FPS when compared to other methods. The specific values were mAP50:95 = 77.84%, AP50 = 99.31%, AP75 = 82.83%, APs = 67.84%, APm = 70.31%, APl = 90.04%, and FPS = 22.3. (4) RT-DETR achieves the best performance on the APm metric (APm = 74.55%), and its other accuracy metrics are better than those of other Transformer-based methods by approximately 0.25% to 1.96%. (5) Compared to RT-DETR, the proposed method improves mAP50:95 by approximately 10.4%, with specific improvements in AP50, AP75, and APs. These accuracy metrics fall within the range of 0.25% to 2.80%, while maintaining similar inference speed (FPS = 23.2, compared to FPS = 22.3 for RT-DETR), reflecting its enhanced efficiency. (6) Our method achieves higher accuracy than YOLOv10-l (mAP = 77.49%) and YOLOv8-l (mAP = 77.49%). The improvements in AP50, AP75, and APl range from 0.30% to 1.00%, while maintaining competitive FPS. Despite the higher speed of YOLOv8-l (FPS = 20.8) and YOLOv10-l (FPS = 32.2), the proposed method excels in high-precision tasks, making it more suitable for applications requiring both speed and accuracy.

By evaluating the network using different pre-trained weights from various self-supervised learning methods, as shown in Table 5, the results indicate that our proposed method outperforms the other methods across several key metrics. Through the *t*-test analysis between MVSSL and the different self-supervised learning methods (mainly analyzing the mAP metric), the t-statistic values were calculated to be in the range [2.98, 20.93] and the *p*-values in the range [0.0004, 0.017]. Since the *p*-value is less than 0.05, it indicates that MVSSL has a statistically significant advantage. Further analysis of the mAP performance reveals that our method achieves the highest mAP of 77.84%, an AP50 of 99.34%, and an AP75 of 82.83%. In comparison, methods such as BYOL, SimSiam, SimMIM, BEIT, and MoCoV3 perform lower across these metrics. For example, BYOL has an mAP of 67.24%, SimSiam achieves 73.45%, and BEIT reaches 75.83%. The proposed method demonstrates a significant improvement, especially in the mAP and AP75, outperforming the baseline methods by approximately 3–10%. These results highlight that integrating novel techniques and optimizations contributes to enhanced feature extraction capabilities in our method.

For the DS-head module, while lightweight calibration techniques, such as Temperature Scaling, offer an elegant and computationally efficient way to align predicted probabilities, their scope is primarily limited to post hoc adjustment of aleatoric uncertainty. In the specific context of railway inspection, however, the challenge often lies in epistemic uncertainty—where subtle texture loss under complex backgrounds can lead to conflicting evidence. The DS-head is integrated to address this by facilitating evidential reasoning directly during the inference process. By modeling basic probability assignments (BPAs), it provides a more nuanced mechanism for resolving detection conflicts and suppressing false positives. Given the safety-critical nature of infrastructure monitoring, the enhanced reliability offered by this evidential approach provides significant practical value that complements the benefits of traditional calibration.

**Figure 9:**
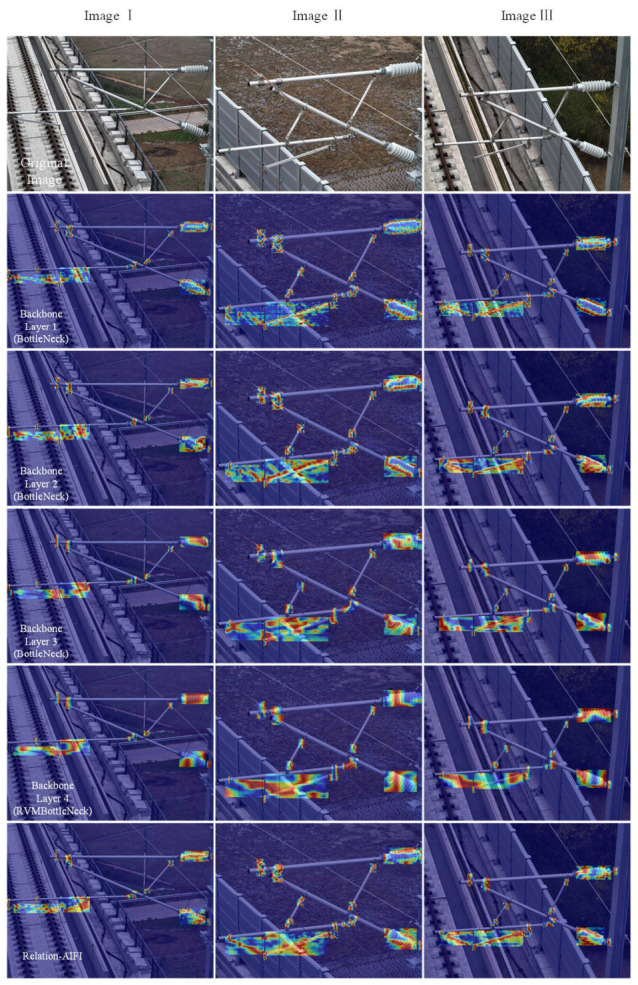
The visualization of a heatmap in different layers.

Based on the experiments conducted, we performed a heatmap visualization analysis to illustrate the impact of the proposed VRM Bottleneck and Relation-AIFI layers on the semantic-level information within the network. Specifically, we visualized the feature outputs of these two critical layers, as shown in Figure 9. From the heatmap visualization, the following conclusions can be drawn: (1) the proposed method effectively enhances attention to key regions of catenary support components through the VRM Bottleneck and Relation-AIFI layers; (2) the Relation-AIFI module improves the network’s ability to focus on spatially dense and geometrically significant regions of catenary components. These observations further validate the effectiveness and rationality of the proposed framework in handling complex scenarios and improving detection performance.

***(3) Visualization of detection results:*** To effectively demonstrate the detection capabilities of the proposed method, visual analysis experiments were conducted on catenary support components (CSCs) across a variety of outdoor settings. The evaluation focused on the method’s ability to maintain robustness when faced with complex and diverse backgrounds captured by UAVs, including challenging features such as bridges, railway tracks, vegetation, and other surrounding structures.

**Figure 10:**
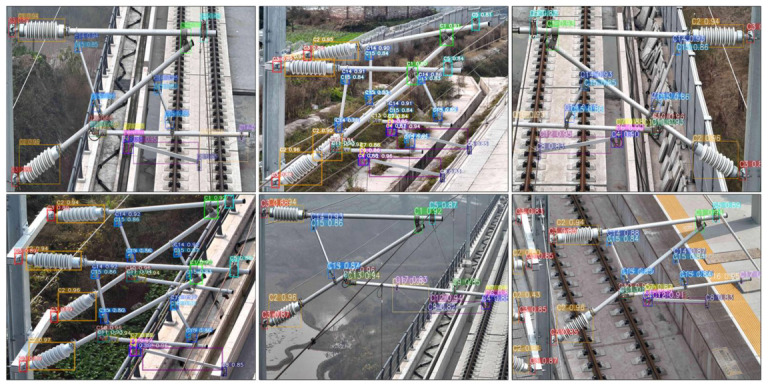
The visual analysis of SPR-DETR.

As shown in Figure 10, six prediction images demonstrate the detection performance of the proposed method in different outdoor scenarios. The results show that the method can accurately detect and identify CSCs even in complex and cluttered backgrounds. In particular, the method performs exceptionally well in detecting components with significant scale variations, such as insulators and support sleeve screws. This demonstrates the robustness and adaptability of the method to address real-world challenges, including multi-scale variations and complex environmental conditions, making it highly suitable for UAV-based inspection applications.

**Figure 11:**
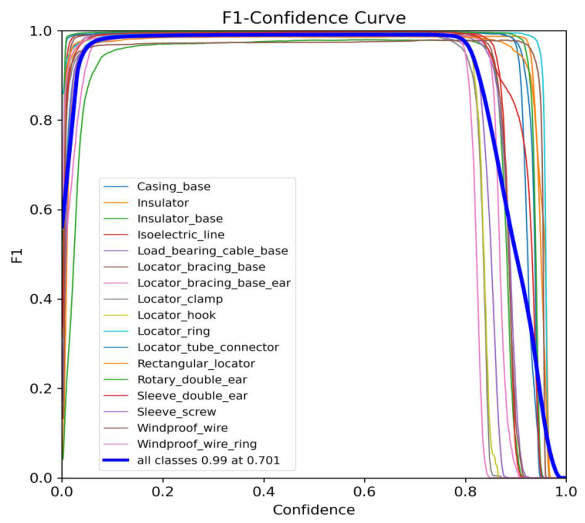
The F1 curves of CSCs.

We also analyzed the F1 curves. As shown in Figure 11, the F1-confidence curves for 17 components are plotted under an IoU threshold of 0.5. The method achieves a peak F1 score of 0.99 at a confidence threshold of 0.7, demonstrating an optimal balance between precision and recall. Notably, the trends remain consistent across all components.

**Figure 12:**
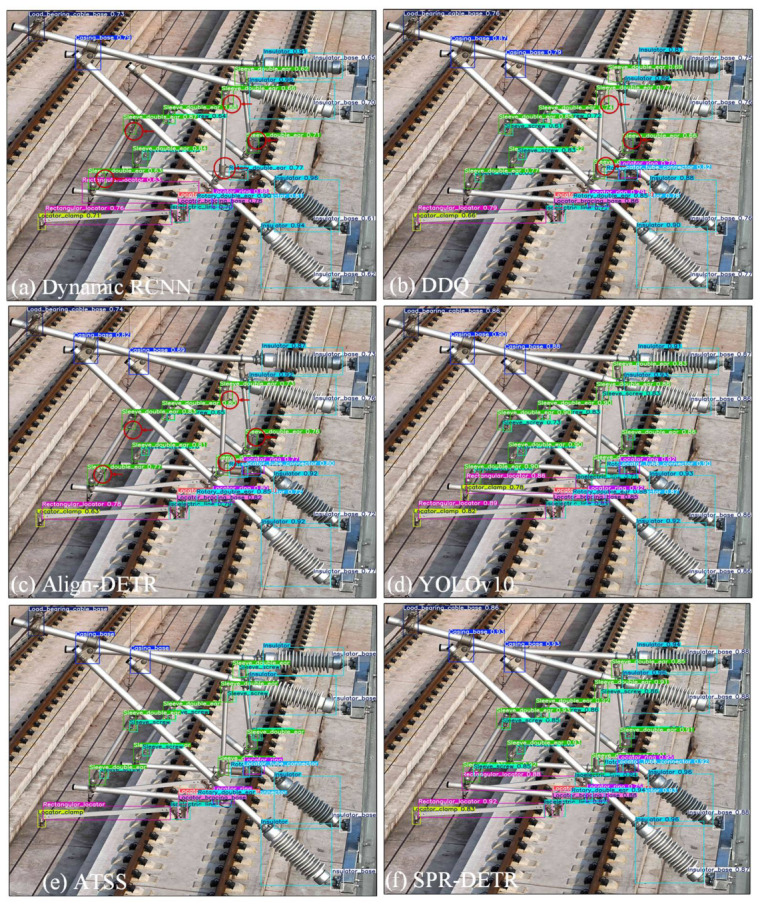
The visual analysis with different methods.

To further validate the superiority of the proposed method, we conducted additional visualization experiments using six different detection methods. As shown in Figure 12, the letters (a) to (f) correspond to the detection results of Dynamic R-CNN, Align-DETR, ATSS, DDQ, YOLOv10, and SPR-DETR, respectively, on the same image. To ensure fairness in the evaluation, the detectors selected for comparison represent the best-performing models among their respective types. Red arrows in the figure highlight the regions where catenary support components were missing missed by the detectors. Based on the visualization results, the following observations can be made: (1) Both the Dynamic R-CNN (a) and ATSS (c) exhibited missed detections for sleeve screws and sleeve double ear components. Specifically, the Dynamic R-CNN failed to detect five sleeve screws and one sleeve double ear, while ATSS missed four sleeve screws and one sleeve double ear. (2) Align-DETR (b) and DDQ (d), as DETR-based models, detected only a few sleeve screws, with most sleeve screws still missed. (3) YOLOv10 (e) and SPR-DETR (f) demonstrated stable performance, successfully detecting all catenary support components without significant omissions. These results indicate that many existing detection methods face considerable challenges when detecting small-sized components, particularly sleeve screws, especially when significant scale variations are present among targets. In contrast, the proposed method exhibits robust detection capabilities across components of various sizes, providing intuitive and strong evidence of its effectiveness in addressing small object detection and multi-scale challenges.

The issues of missed detections mentioned above may lead to the following problems: (1) We noted that some detection methods suffer from missed detections of small components (e.g., sleeve screws), which may lead to severe consequences in real-world applications. In railway inspections, missing key components can delay maintenance, thereby affecting railway safety and operational efficiency. For instance, missed sleeve screws may cause loosening or damage to the railway track, increasing safety hazards. (2) For users relying on these detection results (such as railway inspectors or maintenance personnel), detection errors may impact on their maintenance decisions, thereby affecting the overall safety and efficiency of the railway system. Especially under extreme weather and complex conditions, misclassification may lead to misjudgment of the equipment’s true condition, causing potential hazards to remain undetected and unresolved. (3) Based on the comparison of various detection methods, we analyzed the impact of false positives and false negatives on final decisions. In practical applications, accurate detection results are crucial for assessing component health and formulating maintenance plans. Any errors or omissions may increase maintenance costs and, in extreme cases, lead to equipment failures or accidents.

**Figure 13:**
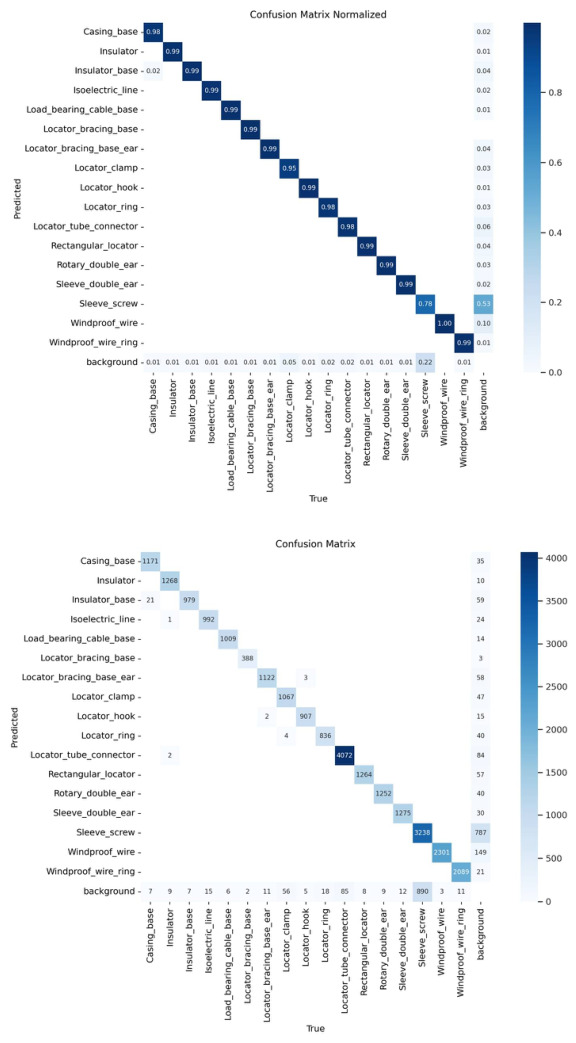
Normalized confusion matrix of the proposed SPR-DETR on the CSC dataset.

To provide a more granular assessment of the model’s performance beyond aggregate metrics, this study introduced a confusion matrix (Figure 13) to analyze inter-class misclassifications and background interference. The results demonstrate that SPR-DETR achieves exceptional precision across the majority of catenary support components, with 15 out of 17 categories reaching diagonal accuracy exceeding 98%. This high level of inter-class discriminability explains why the F1-confidence curves in Figure 11 exhibit highly consistent and nearly overlapping trajectories, validating the model’s robust feature extraction capabilities for components with stable geometric structures.

However, the confusion matrix also reveals specific limitations when handling extreme multi-scale targets. The primary detection failure is concentrated in the Sleeve_screw category: due to its micro-scale size and susceptibility to texture loss under complex railway backgrounds, approximately 22% of actual targets are misidentified as background (Figure 12). This lack of pixel-level features constitutes the main bottleneck for current detection performance. Additionally, a marginal misclassification (0.02) between the Insulator_base and Casing_base reflects slight semantic ambiguity between components with similar metallic textures and compact spatial arrangements. This in-depth failure analysis not only confirms the advantages of SPR-DETR in modeling complex topological relationships but also provides a clear roadmap for future improvements, such as integrating super-resolution enhancement and lightweight operators to optimize micro-object detection.

To evaluate detection accuracy under visually degraded conditions, we selected a single dominant control parameter for each environment type: brightness scale for low-light, fog alpha for dense fog, and noise intensity (value) for heavy rain.

The results are summarized in Table 6. Across all three conditions, we observed clear drops in both mAP and APs as the selected parameter approached or exceeded a critical threshold: (1) when the brightness scale dropped below 0.5 (set to 0.40), mAP dropped to 54.6% and APs to 51.4%; (2) when fog alpha exceeded 0.40 (set to 0.45), mAP dropped to 56.8% and APs to 52.7%; (3) when noise intensity exceeded 350 (set to 400), mAP dropped to 55.2% and APs to 49.6%. These results indicate that the detection accuracy declines significantly when visual interference reaches certain levels, especially for small objects.

**Figure 14:**
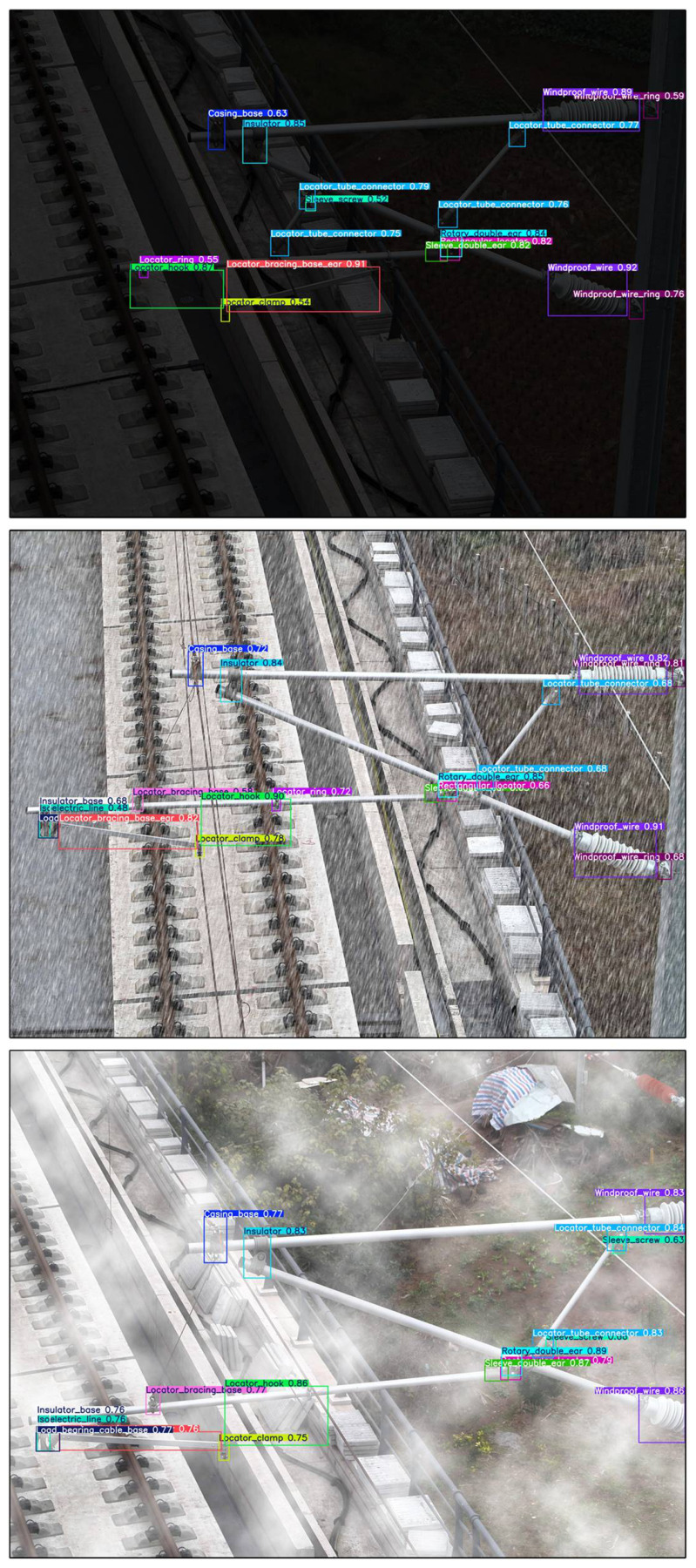
The visual analysis under different weather conditions.

While the above parameter-based analysis identifies performance boundaries under controlled settings, it is also important to assess the model’s behavior under broader, real-world environmental variations. To this end, we conducted experiments using augmented datasets simulating low-light, foggy, and rainy conditions. The model’s detection performance across these scenarios is summarized in Table 7 and visually illustrated in Figure 14.

As shown in Table 7, in terms of small-object detection performance (APs metric), the model also demonstrates strong adaptability under different environmental conditions. Specifically, under foggy conditions (UAV-fog), the highest small-object detection accuracy is achieved, with an APs of 65.28%; under rainy conditions (UAV-rain), the APs reaches 62.45%; and under low-light conditions (UAV-dark), the APs slightly decreases to 59.23%. Although extremely poor lighting imposes certain challenges on small-object detection, overall, the APs values across different weather scenarios remain around or above 60%, further validating the model’s robust capability for detecting small-scale objects in complex environmental conditions. This is further supported by *t*-test analysis, which compared the performance of the model under these different conditions. The t-statistic values were found to be in the range [2.80, 7.46], with *p*-values between [0.0007 and 0.0118]. Since all *p*-values are below 0.05, the results confirm statistically significant differences in the model’s performance across the different weather conditions, reinforcing the model’s strong adaptability and effectiveness in detecting small objects across a variety of challenging environments. The detection performance under these different conditions is visually illustrated in Figure 14.

***(4) Comparison of MVSSL Pre-training Strategies under Different Labeled Data Volumes:*** To verify the effectiveness of the proposed multi-view self-supervised learning (MVSSL) pre-training strategy in alleviating the label scarcity problem in railway inspection scenarios, we designed a series of ablation experiments. The experiments mainly compared the differences in detection accuracy (mAP) of the MVSSL pre-trained model under different labeled data ratios.

As shown in Table 8, the experimental results for the demonstration of saturation at 1/3 labeled data show an obvious logarithmic growth trend. Notably, when the labeling ratio increases from 33.3% to 100%, the mAP only rises by 0.14% (from 77.84% to 77.98%). This result rigorously demonstrates that with the support of MVSSL pre-trained weights, SPR-DETR only requires one-third of the manual labeling resources to capture almost all key discriminative features of catenary components. Accordingly, the proposed method can effectively reduce approximately 66.7% of manual annotation workload while maintaining detection accuracy. Robustness under extremely low labeled data ratios (5–10%): In response to the reviewer’s concern regarding extreme data scarcity, experimental results show that the model still maintains an mAP of 58.45% even with only 5% labeled data. This benefit comes from the robust feature distribution learned by loss adjustment via the Wasserstein distance in the pre-training stage, as well as the profound modeling ability of the Relation Vision Module (RVM) for spatial topological relationships in railway scenarios. Such a powerful structural prior compensates for the severe lack of supervised signals, enabling the model to achieve reliable object localization even in the cold-start stage. Analysis of the performance saturation mechanism: The performance quickly reaches a plateau at the 33% labeling ratio, indicating that the representations learned by MVSSL possess extremely high information density. Through self-supervised pre-training, the backbone network has already acquired view-invariant features of components under diverse road conditions. Consequently, the downstream fine-tuning task mainly serves for category mapping rather than re-learning feature extraction. This characteristic makes SPR-DETR an ideal solution to the dilemma of easy sample collection but high labeling cost in the railway inspection field.

***(5) Quantitative Evaluation of Anomaly Detection:*** We extended our pipeline by incorporating post-localization anomaly detection. Specifically, after identifying key components using SPR-DETR, we introduced the Segment Anything Model (SAM) to perform instance-level segmentation on the localized regions, allowing us to obtain precise contour boundaries of each part [70]. Recent studies have also demonstrated the immense potential of integrating the SAM with invariant normal region prototypes for electrified railway anomaly detection [71]. Based on the segmentation results, we applied the AMI-Net—a state-of-the-art model in industrial visual inspection—for anomaly detection on eight key components: casing base (C1), rotary double ear (C13), insulator (C2), locator base (C6), locator tube connector (C11), locator clamp (C8), sleeve double ear (C14), and sleeve screw (C15).

**Figure 15:**
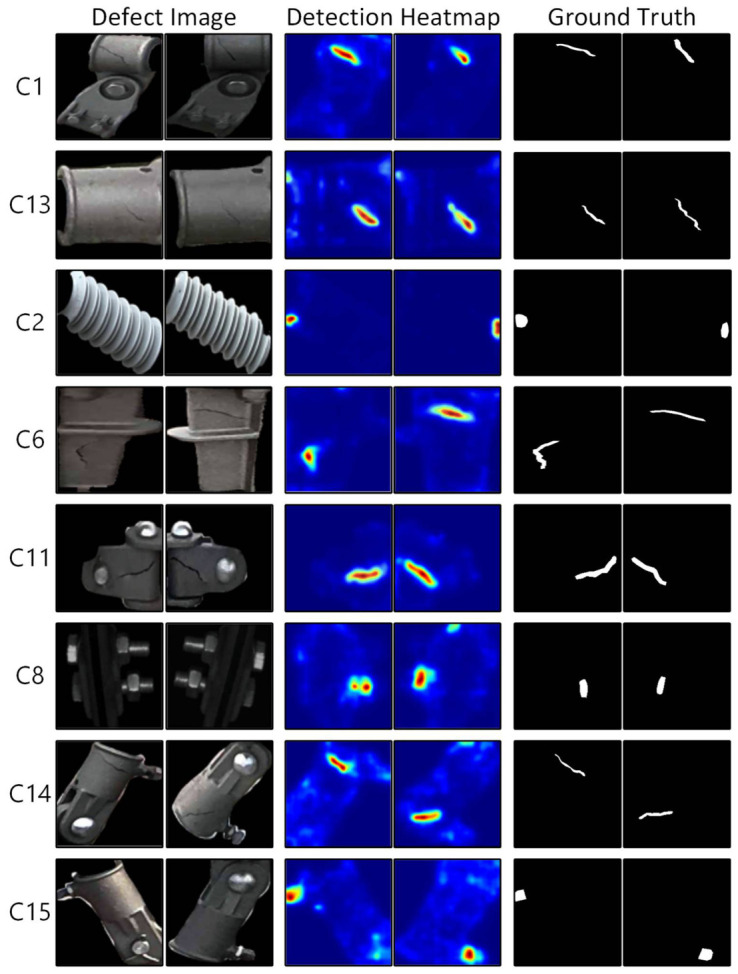
Localization results of AMI-Net on catenary components.

The anomaly detection process focuses on three typical failure modes commonly observed in catenary systems: cracks, looseness, and material loss. As shown in Table 9, AMI-Net achieved high image-level AUROC scores (mostly ranging from 0.92 to 1.0), and the pixel-level metrics also indicated strong boundary-level precision. These results confirm the practical feasibility of deploying our system for industrial anomaly detection. Qualitative detection results based on AMI-Net are illustrated in Figure 15, where the model accurately localizes defect regions across all eight component categories.

**Figure 16:**
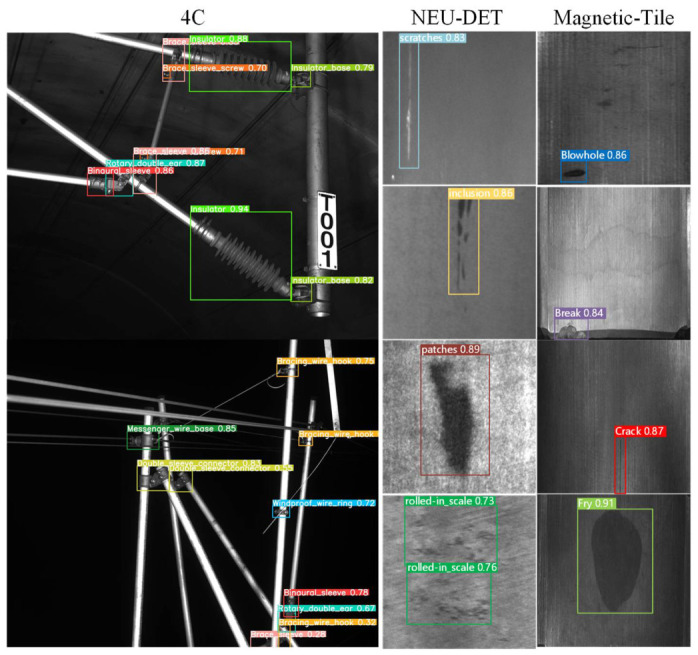
The visualization results of the generalization performance experiment.

***(6) Evaluation of Generalization Capability:*** To assess the generalization capacity of the proposed methodology, experiments were conducted on three datasets with diverse characteristics: NEU-DET [72], Magnetic-Tile [73], and 4C [74]. The 4C dataset includes images of catenary support components captured by a 4C inspection car. It was specifically developed for detecting industrial components within railway catenary systems and contains images of 12 different types of components. The numerical results of the experiments are presented in Table 10, and the results of the generalization experiments are visualized in Figure 16. These results demonstrate the robustness and adaptability of the proposed method across various datasets and scenarios.

Based on the results presented in Table 10, the proposed method achieves an mAP of 72.16%, 67.25%, and 53.82% on the Magnetic-Tile, NEU-DET, and 4C datasets respectively. Additionally, the method demonstrates strong performance in detecting small-scale targets, as indicated by the APs metric. These results highlight the method’s robust generalization ability and its potential for effective application across a variety of object detection tasks. The t-statistic values ranged from [2.64 to 17.16], with *p*-values between [0.008 and 0.0295]. Since all *p*-values are below 0.05, the results confirm that the differences in performance across the datasets are statistically significant, reinforcing the model’s strong adaptability and effectiveness in detecting small objects across diverse conditions.

The performance gap between the self-built UAV dataset and the 4C dataset (53.82% mAP) reveals a limitation in cross-scene generalization. The core reason lies in the geometric representation discrepancy caused by inconsistent imaging perspectives. While the UAV (DJI M30T) provides a top-down perspective, the 4C system employs a bottom-up view. For components sensitive to observation angles, such as specific fasteners and support connectors, their features in the 4C images may undergo significant deformation or even become invisible compared to the pre-training representations. To alleviate this, future work will explore view-invariant feature learning through adversarial domain adaptation and more rigorous geometric data augmentation to bridge the domain gap between different inspection platforms.

## 4. Limitations and Future Work

Despite the superior performance of SPR-DETR, some limitations warrant further investigation. First, the proposed distribution-aware loss utilizes the Wasserstein distance as a static scaling factor. While this suppresses noise, it may inadvertently weaken the gradients for hard samples with extreme environmental variations, potentially leading to sub-optimal representation in rare scenarios. Future research will focus on developing a dynamic weight annealing strategy to balance feature alignment and distribution robustness. Second, the computational complexity of the Relation Vision Module (RVM) and the batch-wise correlation matrix calculation (158.8G FLOPs) remains high for real-time deployment on resource-constrained UAV platforms. Integrating lightweight operators such as coordinate-aware attention and knowledge distillation techniques represents a promising direction to optimize the trade-off between detection accuracy and inference speed.

## 5. Conclusions

This paper proposes an efficient object detector for detecting catenary support components that integrate various innovative strategies. To validate the effectiveness of this method, we conducted qualitative analysis, visualization, comparison, and generalization tests. The following conclusions can be drawn from the experimental results: (1) The proposed SPR-DETR method (ours) achieves an mAP50:95 of 77.84%, APs of 67.84%, APm of 70.31%, and APl of 90.04% on the UAV dataset, demonstrating strong multi-scale detection capabilities, particularly for small-scale objects. (2) Through visualization experiments, it can be observed that the proposed method performs well in detection and can effectively detect CSCs in complex backgrounds. (3) Generalization tests on multiple datasets (NEU-DET, Magnetic-Tile and 4C) verify the effectiveness of the RVM, Relation-AIFI module, and DS-head across different data sources. The results demonstrate the method’s strong cross-domain adaptability. (4) Comparison with classical methods (such as YOLOv10, YOLOv8, and ATSS), as well as DETR methods, shows that the inclusion of the RVM, Relation-AIFI module, and DS-head in SPR-DETR leads to better performance in mAP50:95, FPS, and Pa, particularly in high-precision tasks, while maintaining an inference speed comparable to other methods.

However, it can be seen from the experimental results that the proposed method still has some limitations.

(1) The current data analysis process is conducted offline, resulting in a certain delay in data processing and response, which affects its real-time applicability in practical monitoring scenarios.

(2) The model does not yet incorporate semantic-level segmentation tasks, which are important for achieving finer-grained recognition of components in complex environments.

In practical high-speed railway inspection scenarios, UAVs mainly serve as mobile sensing devices and undertake the tasks of high-frequency image acquisition and real-time data transmission. In future work, we plan to offload complex computations such as object detection and in-depth analysis to dedicated analysis units deployed in automated UAV hangars or edge computing nodes along railway lines via high-bandwidth wireless links such as 5G and microwave communication.

## Figures and Tables

**Table 1:** The hyperparameter settings of the optimizer.

Hyperparameter	Settings
Optimizer	Adam
Weight decay	0.0001
Momentum	0.9
Initial learning rate	0.0001
Final learning rate	0.001
Batch size	64
Epoch	2000

**Table 2:** The parameters of UAV image acquisition.

Parameter	Value/Description
UAV Model	DJI Matrice M30T
Flight Altitude	50 m
Camera—Zoom	48 MP with 20× hybrid optical zoom
Camera—Wide Angle	12 MP with 24 mm equivalent focal length
Image Resolution	6000 × 8000 pixels
Image Bit Depth	12-bit
File Format	JPEG
Sensor Suite	Zoom camera, wide-angle camera
Environmental Conditions	Daylight, normal weather

**Table 3:** Results of the ablation study for the proposed modules.

Module				Accuracy			Speed
RVM	PRE	DS-Head	MVSSL	*AP*_50_	*mAP*	*mAR*	*FPS*
				98.33 (±1.10)	67.42 (±0.85)	98.18	23.2
**✓**				98.72 (±1.20)	71.03 (±1.05)	98.62	22.5
	**✓**			98.64 (±1.05)	70.65 (±0.95)	98.54	22.8
		**✓**		98.51 (±1.30)	69.92 (±1.15)	98.64	22.6
			**✓**	98.86 (±1.15)	70.94 (±1.08)	98.54	22.3
**✓**	**✓**			98.89 (±1.10)	72.74 (±0.98)	99.01	21.8
	**✓**	**✓**		99.01 (±1.25)	73.52 (±1.10)	99.13	22.2
**✓**	**✓**	**✓**		99.12 (±1.00)	74.85 (±0.95)	99.21	21.5
**✓**	**✓**	**✓**	**✓**	99.33 (±0.90)	77.84 (±0.61)	99.41	20.7

**Table 4:** Performance comparison with state-of-the-art detection methods.

Methods	*mAP*	*AP*_50_	*AP*_75_	*AP_s_*	*AP_m_*	*AP_l_*	Pa/M	FLOPs/G	FPS
Dynamic RCNN [60]	59.52 (±1.64)	94.72 (±1.08)	65.90 (±2.4)	58.13 (±1.02)	63.07 (±2.34)	78.74 (±0.78)	41.41	197.7	16.2
Faster RCNN [32]	56.20 (±2.47)	91.52 (±1.23)	62.06 (±2.43)	52.0 (±1.83)	61.8 (±0.68)	75.32 (±2.1)	45.96	235.6	10.1
MFI-YOLO (YOLOv8-l) [61]	67.63 (±2.15)	96.13 (±1.41)	77.49 (±2.12)	62.79 (±1.12)	74.73 (±0.89)	84.11 (±0.65)	43.7	161.1	20.8
YOLOv10-l [62]	71.32 (±1.98)	96.72 (±2.07)	79.15 (±1.11)	63.25 (±1.54)	75.33 (±0.59)	84.06 (±2.47)	25.79	127.3	30.7
DINO [63]	67.21 (±1.67)	96.51 (±0.9)	76.3 (±0.7)	62.06 (±1.59)	72.02 (±1.15)	83.59 (±2.04)	47.56	265.6	15.5
Align-DETR [64]	66.58 (±1.86)	96.01 (±1.53)	76.3 (±1.87)	60.80 (±0.87)	73.60 (±1.28)	83.20 (±0.9)	47.51	253.8	15.1
DDQ (DETR) [65]	65.75 (±2.25)	96.1 (±1.68)	5.2 (±1.38)	62.53 (±2.44)	73.92 (±1.04)	84.10 (±0.51)	65.75	852.5	15.4
SSD [65]	53.84 (±2.11)	90.24 (±0.59)	57.08 (±0.74)	46.57 (±2.05)	57.32 (±2.16)	70.13 (±2.13)	34.10	145.8	21.7
ATSS [66]	58.91 (±1.83)	94.83 (±1.72)	63.11 (±1.49)	58.12 (±2.38)	66.85 (±1.21)	82.34 (±1.91)	32.14	192.4	15.6
RT-DETR [55]	67.44 (±1.54)	96.42 (±0.84)	77.45 (±0.57)	63.28 (±2.29)	74.55 (±1.06)	83.51 (±1.96)	42.14	125.7	23.2
GSINet [40]	73.6 (±0.78)	99.21 (±0.69)	82.51 (±2.46)	66.4 (±1.65)	70.9 (±1.42)	91.6 (±1.94)	23.1	84	28.9
SPR-DETR (ours)	77.84 (±0.61)	99.34 (±0.63)	82.83 (±2.32)	67.84 (±1.73)	70.31 (±1.59)	90.04 (±2.04)	68.18	158.8	20.7

Note: Pa represents the model’s parameters, and its unit is M; FLOPs represent the computational complexity of the model, and their unit is G.

**Table 5:** Comparative experiments for self-supervised learning method.

SSL	*mAP*	*AP*_50_	*AP_s_*	*AP_m_*	*AP_l_*
BYOL [67]	67.24 (±1.23)	98.41 (±0.30)	64.91 (±0.45)	74.12 (±0.52)	84.23 (±0.40)
SimSiam [45]	73.45 (±2.01)	98.92 (±0.35)	66.85 (±0.50)	74.57 (±0.58)	84.61 (±0.38)
SimMIM [46]	72.63 (±1.45)	98.54 (±0.32)	65.94 (±0.48)	73.90 (±0.55)	84.45 (±0.42)
BEIT [68]	75.83 (±1.87)	99.32 (±0.28)	67.02 (±0.40)	**75.81 (±0.50)**	85.24 (±0.45)
MoCoV3 [69]	74.82 (±1.67)	98.91 (±0.31)	66.88 (±0.47)	74.97 (±0.53)	84.80 (±0.43)
MVSSL (ours)	**77.84 (±0.61)**	**99.34 (±0.25)**	**67.84 (±0.38)**	70.31 (±0.60)	**90.04 (±0.35)**

Note: SSL represents the model’s parameters for self-supervised learning, and the bold values indicate the best-performing methods.

**Table 6:** Comparative experiments for self-supervised learning methods under different weather conditions.

Condition	Parameter	Value	Range	Threshold	*mAP*	*AP_s_*
Low Light	Brightness Scale	0.40	0.3–0.7	<0.5	54.6	51.4
Fog	Opacity (Alpha)	0.45	0.25–0.50	>0.40	56.8	52.7
Rain	Noise Intensity (value)	400	100–500	>350	55.2	49.6

Note: Parameter refers to control parameter, Value refers to current value, Range refers to parameter range, and Threshold refers to critical threshold.

**Table 7:** The experiments of different weather conditions.

Dataset	*mAP*	*AP*_50_	*AP_s_*	*AP_m_*	*AP_l_*
UAV-dark	60.43 (±1.23)	95.27 (±0.45)	59.23 (±0.52)	72.65 (±0.68)	81.58 (±0.35)
UAV-rain	62.91 (±1.45)	96.07 (±0.55)	62.45 (±0.60)	74.83 (±0.75)	85.54 (±0.40)
UAV-fog	65.16 (±1.34)	96.32 (±0.50)	65.28 (±0.55)	75.23 (±0.60)	87.75 (±0.45)

Note: Parameter refers to control parameter, Value refers to current value, Range refers to parameter range, and Threshold refers to critical threshold.

**Table 8:** Comparison of MVSSL pre-training strategies under different labeled data volumes.

Different Labeling Ratios	SPR-DETR (MVSSL)-mAP
5%	58.45%
10%	67.20%
20%	73.55%
33%	77.84%
100%	77.98%

**Table 9:** Anomaly detection results in terms of image-/pixel-level auroc and ap on key components.

Component	Image AUROC	Pixel AUROC	Image AP	Pixel AP
Casing base	0.924	0.982	0.873	0.128
Rotary double ear	1.000	0.978	1.000	0.124
Insulator	1.000	0.998	0.999	0.623
Locator base	0.989	0.985	0.985	0.271
Locator tube connector	0.991	0.988	0.984	0.27
Locator clamp	0.992	0.978	0.978	0.142
Sleeve double ear	0.986	0.978	0.978	0.103
Sleeve screw	0.605	0.792	0.363	0.078

**Table 10:** The experiments of generalization performance.

Dataset	*mAP*	*AP*_50_	*AP_s_*	*AP_m_*	*AP_l_*
NEU-DET [72]	67.25 (±1.30)	96.04 (±0.40)	59.03 (±0.50)	67.06 (±0.65)	79.05 (±0.60)
Magnetic-Tile [73]	72.16 (±1.50)	96.15 (±0.35)	64.22 (±0.55)	71.23 (±0.70)	81.00 (±0.65)
4C [74]	53.82 (±1.10)	95.12 (±0.30)	51.16 (±0.45)	59.83 (±0.60)	64.46 (±0.50)

## Data Availability

The datasets used in this study are not publicly available due to confidentiality agreements and legal restrictions. Access to the data is restricted to comply with relevant security and privacy regulations.
